# Taxonomic notes on *Napeanthus* (Gesneriaceae) in Ecuador: neotypification of two species and a new species from the eastern Andean slopes

**DOI:** 10.3897/phytokeys.273.189993

**Published:** 2026-04-16

**Authors:** John L. Clark

**Affiliations:** 1 Marie Selby Botanical Gardens, 1534 Mound St., Sarasota, FL 34236, USA Marie Selby Botanical Gardens Sarasota United States of America

**Keywords:** Andes, biodiversity, Ecuador, Napeantheae, taxonomy

## Abstract

Ongoing field expeditions in Ecuador and herbarium research have resulted in the discovery of a new species and the stabilization of two names in *Napeanthus* (Gesneriaceae). *Napeanthus robustus* Fritsch and *Napeanthus ecuadorensis* Fritsch were both described from specimens housed at the Berlin herbarium that were subsequently destroyed; therefore, neotypes are designated to stabilize the application of these names. A third taxon, *Napeanthus decurrens* J.L.Clark, **sp. nov**., is described as a narrow endemic from the eastern Andean slopes of the Ecuadorian province of Zamora-Chinchipe.

## Abstract (es)

Expediciones recientes de campo en Ecuador, complementadas con la revisión de material de herbario, han permitido el descubrimiento de una nueva especie y la estabilización de dos nombres del género *Napeanthus* (Gesneriaceae). *Napeanthus robustus* Fritsch y *Napeanthus ecuadorensis* Fritsch fueron descritas a partir de ejemplares depositados en el herbario de Berlín; sin embargo, los cuales fueron posteriormente destruidos. Por ello, con el fin de asegurar la correcta aplicación y estabilidad de ambos nombres, aquí se designan neotipos. Asimismo, se describe un tercer taxón, *Napeanthus decurrens* J.L.Clark, **sp. nov**., el cual constituye un endemismo restringido a las laderas orientales de los Andes ecuatorianos, en la provincia de Zamora-Chinchipe.

## Introduction

The genus *Napeanthus* Gardner is the sole genus of the tribe Napeantheae, the smallest tribe within the Neotropical Gesneriaceae (Weber et al. 2013, 2020). Recent phylogenomic studies based on targeted capture of nuclear genes support the placement of Napeantheae as basal within the subfamily Gesnerioideae and sister to all remaining members of the subfamily (Ogutcen et al. 2021).

*Napeanthus* is currently represented by 18 species (Clark et al. 2020; GRC 2026), many of which remain poorly known and underrepresented in herbaria. One of the principal taxonomic challenges in studying *Napeanthus* is the ephemeral nature of its flowers. The corollas are small and delicate, easily abscising and disintegrating from their slender corolla tubes, making accurate documentation dependent upon careful field observations and high-quality photographic records. *Napeanthus* is distributed throughout the Neotropics and is characterized by a basal rosette of leaves and actinomorphic, truncate flowers lacking nectaries. The corolla tubes are typically white or white suffused with blue. The inflorescences commonly bear multiple pairs of persistent, leaf-like bracts and often elongate along the forest floor, in some species reaching nearly one meter in length.

Taxonomic interpretation of *Napeanthus* in Ecuador is complicated by the fact that two names published by Fritsch (1925) were described from specimens housed at the Berlin herbarium that were subsequently destroyed during World War II; therefore, neotypes are selected here to stabilize the application of these two names. Luis Sodiro (1836–1909), a Jesuit priest and one of the most influential botanists working in Ecuador during the late nineteenth century, conducted extensive botanical exploration in western Ecuador, particularly in the vicinity of Santo Domingo and adjacent Andean and lowland regions. His collections formed the basis of numerous early taxonomic treatments and represent some of the earliest systematic botanical documentation of Ecuador’s flora. Revisiting these historical collecting sites and evaluating specimens at the herbaria Universidad Central del Ecuador (Q) and Herbario Padre Luis Sodiro in the Biblioteca Aurelio Espinosas Pólit (QPLS) played an important role in selecting neotypes for two names that correspond to commonly collected species of *Napeanthus* in western Ecuador.

## Materials and methods

Ongoing herbarium research and field expeditions throughout Ecuador since the 1990s have generated numerous collections that are difficult to identify to species. A careful review of the protologues in Fritsch (1925), combined with renewed field investigations in localities historically visited by Sodiro, facilitated the selection of neotypes that correspond to the type localities and are congruent with the original descriptions.

Digital images of live specimens were taken in the field using a Nikon DSLR camera equipped with a Nikon 105 mm macro lens and a Nikon SB-29s ring flash. Morphological observations and measurements were made from live collections, herbarium specimens, alcohol-preserved material, and digital images. The latter were analyzed using the software ImageJ (Schneider et al. 2012).

## Taxonomic treatment

### Napeanthus decurrens

Taxon classificationPlantaeLamialesGesneriaceae

J.L.Clark sp. nov.

366B0CAE-B31D-5E23-B406-F64F4524CBA3

urn:lsid:ipni.org:names:77378701-1

Fig. 1

#### Diagnosis.

Similar to *Napeanthus loretensis* L.E.Skog in its small (< 9 cm long), decurrent leaves, but *N. decurrens* is distinguished by entire leaf margins and erect inflorescences (vs. serrate leaf margins and prostrate inflorescences in *N. loretensis*).

#### Type.

Ecuador. Zamora-Chinchipe • Cantón Nangaritza, Laberinto de las Mil Ilusiones, 1–2 km east of Río Numpatakayma, 4°14'54"S, 78°39'34"W, 1000 m, 14 May 2009, *J.L. Clark 10808* (holotype, SEL [barcode SEL069589]!; isotypes, ECUAMZ, MO, QCNE, US [barcode 01921756]!).

#### Description.

Terrestrial or lithophytic herb with leaves in a basal rosette. ***Leaves*** opposite, equal within a pair, appearing sessile due to the blade being decurrent along the petiole; blades spathulate to broadly obovate, 2–9 × 0.5–2 cm, coriaceous, glabrous, adaxially green, abaxially red to green suffused with red; apex acute; margins entire; secondary veins 3–5 pairs. ***Inflorescences*** erect, 2–5 cm long, usually bearing 1–2 pairs of leafy bracts subtending the pedicels. ***Flowers*** nearly actinomorphic and erect at maturity. Calyx green, glabrous externally and internally; lobes 5, nearly free, fused at the base for 1–2 mm; valvate in bud and spreading at anthesis; lobes nearly equal, broadly oblong with acuminate apices, 3.5–4.5 × 0.5–2.3 mm. Corolla actinomorphic; tube nearly truncate at maturity; lobes deeply divided and nearly free, uniformly white to white suffused with blue; lobes broadly oblong, 5–9 × 3–5 mm; corolla diameter up to 15 mm. ***Androecium*** of 4 erect stamens adnate to the base of the corolla tube; filaments flattened, ca. 3 mm long, white, glabrous; anthers often coherent when immature and spreading at maturity, oblong, dehiscing longitudinally, 1.2–1.4 × 1.2–1.3 mm. ***Gynoecium*** of a superior, broadly oblong ovary, 2.0 mm wide at base; style stout, included, ca. 3 mm long; stigma stomatomorphic. Fruits not observed.

#### Additional specimens examined.

Ecuador. Zamora-Chinchipe • Cantón Nangaritza, parroquia Nuevo Paraíso, Laberinto de Las Mil Ilusiones, 1–2 km east of Río Numpatakayma, 4°14'54"S, 78°39'34"W, 1001 m, 6 Mar 2017, *J.L. Clark, J.A. Mayr & D.A. Neill 15074* (E, ECUAMZ, F, G, MO, MT, NY, QCA, SEL, US); • Cantón Nangaritza, parroquia Nuevo Paraiso, Laberinto de Las Mil Ilusiones, 1–2 km east of Río Numpatakayma, 4°22'2"S, 78°39'39.6"W, 1000 m, 5 Mar 2018, *J.L. Clark 15569* (ECUAMZ, MO, QCA, SEL, US); • Cantón Nangaritza, parroquia Nuevo Paraiso, Laberinto de Las Mil Ilusiones, 1–2 km east of Río Numpatakayma, 4°22'2"S, 78°39'39.6"W, 1000 m, 11 Mar 2019, *J.L. Clark & A. Wilcox 16255* (ECUAMZ, QCA, SEL).

#### Phenology.

Collected with flowers in March and May.

#### Etymology.

The specific epithet *decurrens* refers to the leaf blades that are decurrent along the petiole, giving the leaves a nearly sessile appearance.

#### Distribution.

*Napeanthus decurrens* is currently known only from the type locality in Zamora-Chinchipe Province, Ecuador, near the Río Numpatakayma, a tributary of the Río Nangaritza. The area, known locally as the Laberinto de las Mil Ilusiones, is characterized by maze-like sandstone rock formations reaching up to ca. 20 m in height. The site is privately managed and accessible primarily by boat, and it is visited by tourists and naturalists staying at nearby Cabañas Yankuam.

#### Comments.

**Figure 1.**
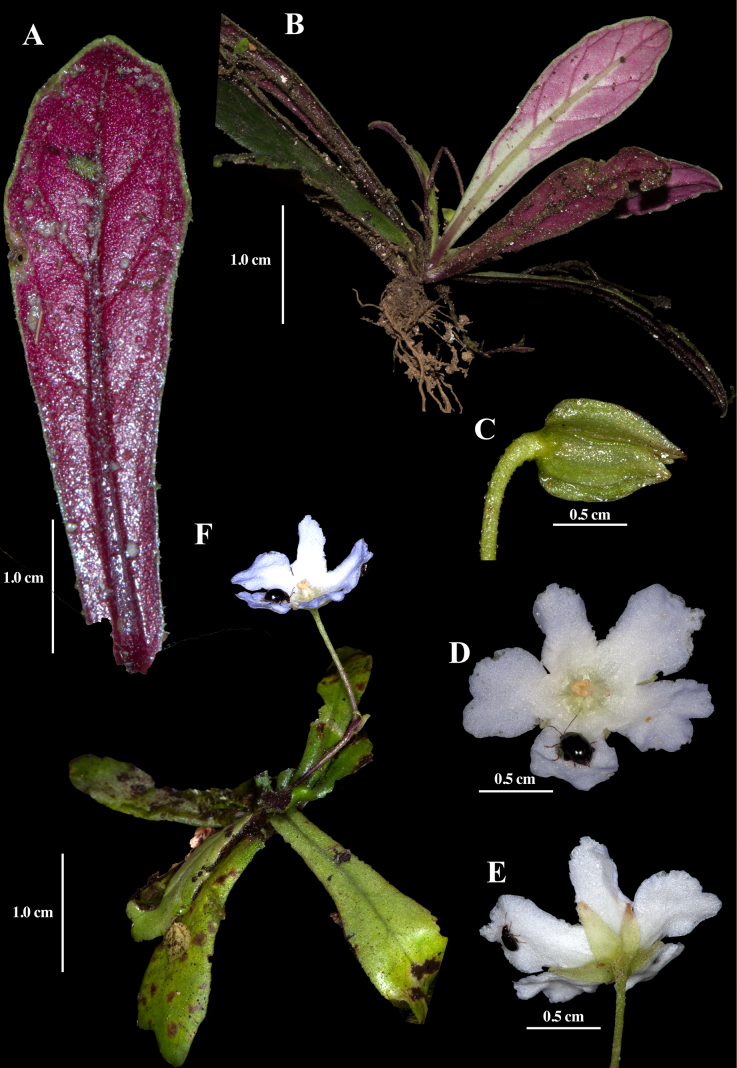
Field images of *Napeanthus decurrens* J.L. Clark. **A**. Abaxial leaf surface; **B**. Habit; **C**. Calyx; **D**. Front view of flower; **E**. Rear view of flower. **F**. Habit; (**A** from *J.L. Clark et al. 10808*; **B** from *J.L. Clark et al. 15074*; **C–E** from *J.L. Clark et al. 10808*). Photos by J.L. Clark.

The combination of small stature (Fig. 1), erect inflorescences (Fig. 1F), and entire leaf margins (Fig. 1A) readily distinguishes *Napeanthus decurrens* from all other congeners. Most individuals were found growing as lithophytes on limestone rocks. The leaves form a compact basal rosette, as in most *Napeanthus*, but in this species the plants appear acaulescent (Fig. 1B, F). The undersurfaces of the leaves are typically red, a coloration that is unusual in *Napeanthus*, where most species have uniformly green leaves. Rarely, the leaves of *N. decurrens* are entirely green or green suffused with red. Another morphologically similar species is *N. loretensis* (Skog 1974), but *N. decurrens* differs by entire leaf margins (Fig. 1A) and erect inflorescences (Fig. 1F). In contrast, *N. loretensis* has serrate leaf margins, and prostrate inflorescences.

### Napeanthus ecuadorensis

Taxon classificationPlantaeLamialesGesneriaceae

Fritsch, Akad. Wiss. Wien Sitzungsber., Math. Naturwiss. Kl., Abt. 1, 134: 125–126 (1925).

113B6A37-29A0-56BB-9E93-E06A26177C8C

Fig. 2

#### Holotype.

Ecuador • serus fl. Peripae [upper Río Peripá basin or western Andean foothills near Santo Domingo], 350 m, Aug 1882, *L. Sodiro 119/57* (B, destroyed); neotype designated here: Ecuador • Santo Domingo de los Tsáchilas, 0°28'38.1"S, 79°11'22.4"W, 560–600 m, 12 Jul 2022, *J.L. Clark, L. Hooge, C. Restrepo, R. Clark & E. Muñoz 16775* (neotype: QCA!; isoneotypes: AAU, BM, BRIT, E, F, FLAS, G, MO, NY, QCA, SEL, US).

#### Comments.

A Sodiro specimen at the Universidad Central del Ecuador (Q) bears the same collection number cited in the protologue; however, that specimen is from a higher elevation and dated October 1882. In contrast, the protologue indicates August 1882 and an elevation of 350 m, which is consistent with material commonly collected throughout the environs of Santo Domingo in western Ecuador where Sodiro frequently worked. There are several specimens from the Herbario Padre Luis Sodiro (QPLS) that are conspecific with *N. ecuadorensis*, but none of them correspond to the locality information and dates from the protologue (Fritsch 1925).

**Figure 2.**
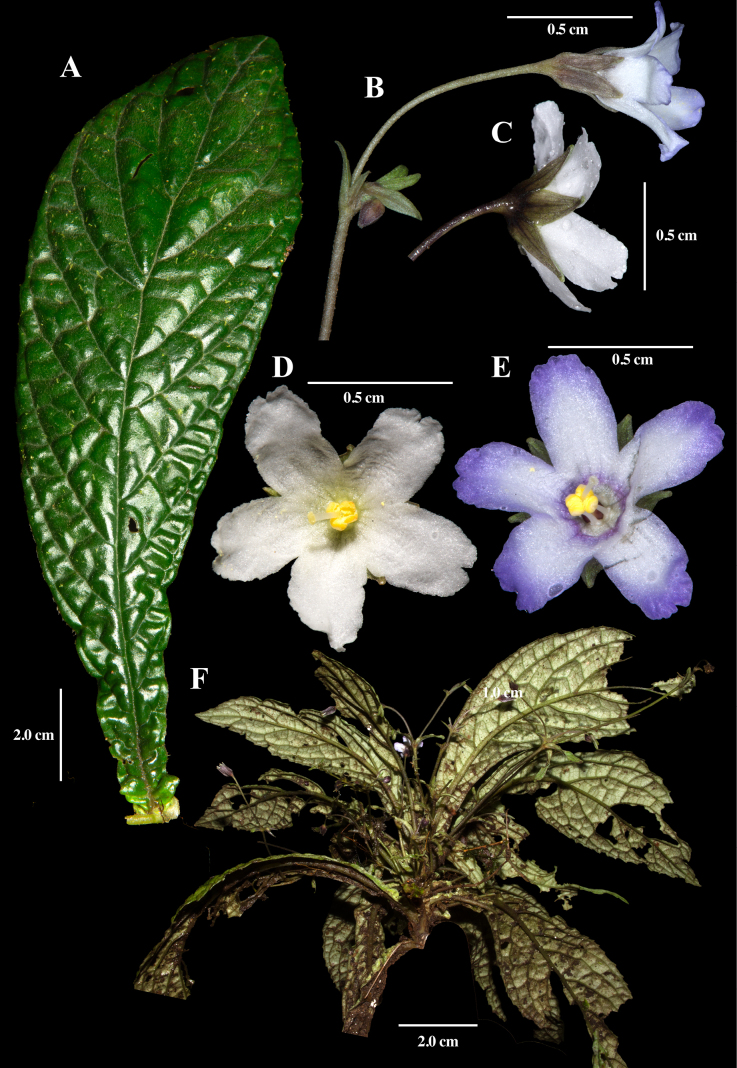
Field images of *Napeanthus ecuadorensis* Fritsch. **A**. Adaxial leaf surface; **B**. Flower with floral bracts; **C**. Lateral view of flower. **D, E**. Front view of flowers; **F**. Habit; (**A, D** from *J.L. Clark et al. 16645*; **B, F** from *J.L. Clark et al. 16293*; **C** from *J.L. Clark et al. 16775*). Photos by J.L. Clark.

**Figure 3.**
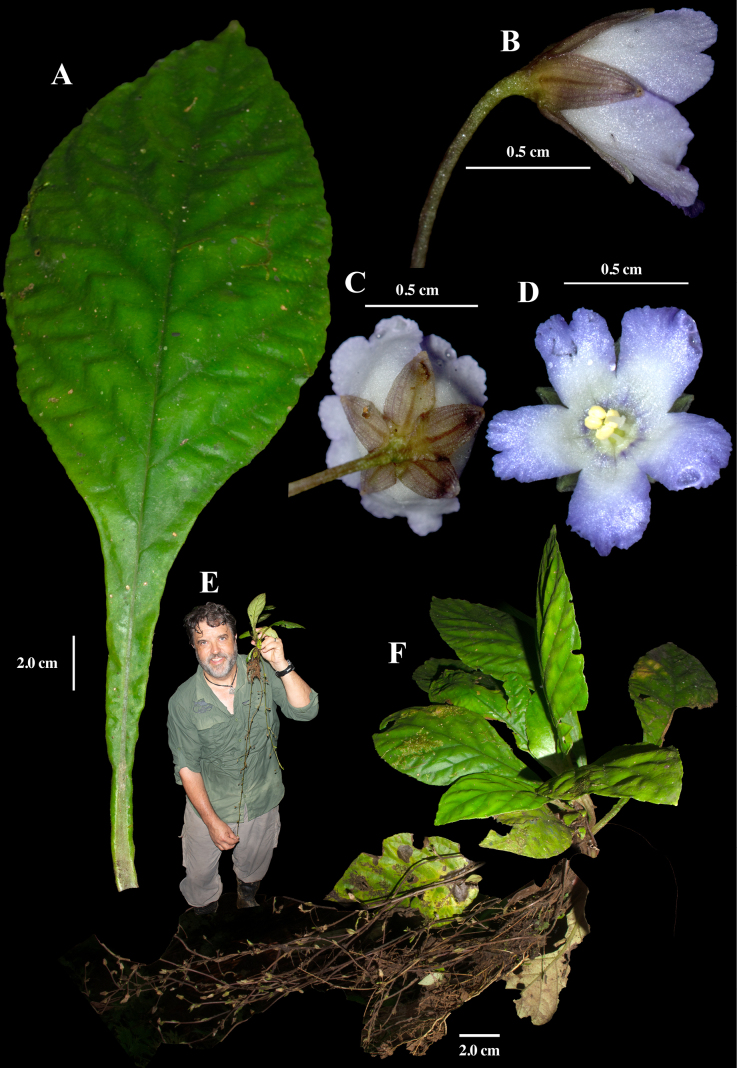
Field images of *Napeanthus robustus* Fritsch. **A**. Adaxial leaf surface; **B**. Lateral view of flower and pedicel; **C**. Rear view of flower; **D**. Front view of flower; **E**. Elongate inflorescence with author shown for scale; **F**. Habit; (**A, E, F** from *J.L. Clark et al. 19867*; **B–D** from *J.L. Clark et al. 12273*). Photos by J.L. Clark.

Leaves of *Napeanthus ecuadorensis* are bullate (Fig. 2A), and the inflorescences are erect or prostrate (Fig. 2F). Another vegetatively distinctive feature is the blade broadly decurrent along the petiole, giving the leaves a broadly spathulate appearance (Fig. 2A). The inflorescences are fewer-flowered and markedly shorter than those of *N. robustus* (Fig. 3), typically less than 20 cm long, whereas those of *N. robustus* often exceed 90 cm.

### Napeanthus robustus

Taxon classificationPlantaeLamialesGesneriaceae

Fritsch, Akad. Wiss. Wien Sitzungsber., Math. Naturwiss. Kl., Abt. 1, 134: 122–123 (1925).

D25D33F0-A4B7-5BE0-9AC9-11827A06934A

Fig. 3

#### Holotype.

Ecuador • in silvis tropicis prope S. Domingo [tropical forests near Santo Domingo], 360 m, Aug 1875, *L. Sodiro 119/58* (B, destroyed); neotype designated here: Ecuador • Santo Domingo de los Tsáchilas, 0°29'13.4"S, 79°11'0.4"W, 840–850 m, 13 Jul 2022, *J.L. Clark, L. Hooge, C. Restrepo, R. Clark & N. Zapata 16831* (neotype: QCA!; isoneotypes: MO, NY, QCA, SEL [barcode: SEL091047]!, US).

#### Comments.

This is one of the most frequently collected species of *Napeanthus* in western Ecuador and is relatively large. Fritsch (1925) noted that it was the most robust and largest species of *Napeanthus* known at the time, consistent with field observations, particularly in the vicinity of Santo Domingo where *N. robustus* and *N. ecuadorensis* occur sympatrically. Sodiro frequently collected near Santo Domingo, where this species remains common in shaded forests. There are several specimens from the Herbario Padre Luis Sodiro (QPLS) that are conspecific with *N. robustus*, but none of them correspond to the locality information and dates from the protologue (Fritsch 1925).

The leaves are large, slightly decurrent along the petiole, with a well-defined petiole and obovate blades, matching the description provided in the protologue (Fritsch 1925). One of the most distinctive features of this taxon is the elongate inflorescences that grow prostrate along the forest floor. These inflorescences often exceed 80 cm in length—more than twice the height of the plant (Fig. 3E)—and are frequently concealed in the understory but readily observed during specimen collection.

## Supplementary Material

XML Treatment for Napeanthus decurrens

XML Treatment for Napeanthus ecuadorensis

XML Treatment for Napeanthus robustus

## Additional information

### Artificial Intelligence (AI) use

The author accepts full responsibility for the content of the manuscript, including the disclosure of any use of AI.

No AI tools were used in the preparation of this manuscript.

### Author ORCIDs

J.L. Clark https://orcid.org/0000-0002-1414-6380

### Data availability

All of the data that support the findings of this study are available in the main text.
